# Interleukin-6-to-lymphocyte ratio as a novel prognostic factor in patients with severe fever with thrombocytopenia syndrome: a discovery and validation study

**DOI:** 10.3389/fimmu.2026.1780148

**Published:** 2026-03-25

**Authors:** Wan Peng, Jingxia Wang, Ruihua Zhang, Zhouling Jiang, Ranran Wang, Mengxin Liu, Ling Lin, Jianping Duan, Zhihai Chen

**Affiliations:** 1 National Key Laboratory of Intelligent Tracking and Forecasting for Infectious Diseases, Beijing Ditan Hospital, Capital Medical University, Beijing, China; 2 Department of Infectious Diseases, Yantai Qishan Hospital, Yantai, China; 3 Department of Infectious Diseases, Qingdao Sixth People’s Hospital, Qingdao, China

**Keywords:** *Bandavirus dabieense*, interleukin-6, lymphocyte, mortality, severe fever with thrombocytopenia syndrome

## Abstract

**Background:**

Severe fever with thrombocytopenia syndrome (SFTS) is an emerging infectious disease, characterized by cytokine storm and immune suppression. The interleukin-6-to-lymphocyte ratio (IL-6/LY) has demonstrated prognostic value in viral infections with dysregulated host immune responses. This study aimed to evaluate the prognostic value of IL-6/LY for early mortality in patients with SFTS.

**Methods:**

This multicenter cohort included 323 patients at Yantai Qishan Hospital (January 2022-December 2024) as the training cohort and 172 patients at Qingdao No. 6 People’s Hospital (January 2023-October 2025) as the validation cohort. Univariable and multivariable logistic regression identified independent predictors of mortality. Prognostic performance was assessed using the area under the receiver operating characteristic (ROC) curve, and clinical benefit was evaluated by decision curve analysis (DCA). Survival was analyzed using the Kaplan–Meier method with the log-rank test.

**Results:**

A total of 495 patients were included in the study, with mortality rate of 21.21% (105/495). Multivariate logistic regression analysis identified DBV RNA level, age, and IL-6/LY as independent predictors of fatal outcome in SFTS. Compared with other predictors, IL-6/LY showed the strongest predictive performance for mortality in the training cohort (AUC = 0.839, 95% CI: 0.792–0.886; P < 0.001) and was externally validated with excellent discrimination (AUC = 0.812, 95% CI: 0.735–0.890; P < 0.001). Patients with elevated IL-6/LY levels (≥ 1.77) had a significantly lower survival probability than those with lower IL-6/LY (log-rank P < 0.001). DCA demonstrated great clinical benefit for IL-6/LY in both the training and external validation cohorts.

**Conclusion:**

IL-6/LY as a novel marker associated with immune dysregulation shows strong independent prognostic value for mortality in SFTS.

## 1 Introduction

Severe fever with thrombocytopenia syndrome (SFTS) is an emerging tick-borne infectious disease caused by Bandavirus dabieense (DBV). Since its first identification in China in 2009, SFTS has rapidly expanded across East and Southeast Asia, with confirmed cases reported in South Korea, Thailand, Vietnam, and Myanmar (1–5). In addition to vector−borne transmission, person−to−person spread through direct contact with the blood or bodily fluids of infected individuals has been reported, further heightening public health concern (6, 7). The major clinical manifestations of SFTS include fever, thrombocytopenia, leukopenia, hemorrhage, gastrointestinal symptoms, and multiple organ dysfunction, with a high case fatality rate ranging from 5% to 30% (8, 9). However, no specific antiviral agents or licensed vaccines are currently available for DBV infection. Consequently, early identification of patients at high risk of disease progression is essential for improving clinical outcomes and reducing mortality.

Previous studies have shown that a variety of biomarkers, including the neutrophil−to−lymphocyte ratio (NLR) (10), platelet−to−lymphocyte ratio (PLR) (11), C-reactive protein-to-albumin ratio (CAR) (12), and inflammatory burden index (IBI) (13) can serve as indicators of adverse outcomes in patients with SFTS. Nevertheless, increasing evidence suggests that cytokine storm and immune dysregulation are major contributors to the rapid clinical deterioration observed in severe cases (14, 15). Conventional composite markers, which do not account for cytokine levels, may fail to adequately reflect the complex immune-inflammatory imbalance underlying disease progression. The interleukin−6 to lymphocyte ratio (IL-6/LY) integrates serum IL-6 levels with lymphocyte counts as a composite marker of inflammatory and immune status. Elevated IL−6/LY has been identified as an independent predictor of in−hospital mortality and multiple organ dysfunction syndrome in patients with severe COVID−19 (16), suggesting its potential applicability to other infectious diseases with hyperinflammatory responses. However, its prognostic significance in SFTS has not been explored. Therefore, this study is the first to investigate the association between IL−6/LY and disease severity in patients with SFTS and to evaluate its predictive value for early mortality, which could facilitate timely clinical decision-making and ultimately improve patient outcomes.

## 2 Methods

### 2.1 Study design and participants

In this multicenter cohort study, 323 patients with SFTS who were hospitalized at Yantai Infectious Disease Hospital from January 2022 to December 2024 were included in the training cohort, while 172 patients admitted to Qingdao No. 6 People’s Hospital between January 2023 and October 2025 were included in the external validation cohort. The inclusion criteria were as follows: (1) aged ≥ 18 years old; and (2) laboratory-confirmed SFTS defined by a positive test for DBV nucleic acid, or isolation of DBV from clinical specimens, or seroconversion of DBV-IgG (defined as a ≥ 4-fold increase in antibody titer between acute- and convalescent-phase sera). The exclusion criteria were as follows: (1) patients with other poorly controlled acute or chronic diseases; (2) patients with acute infections caused by other pathogens; and (3) patients with incomplete clinical data.

### 2.2 Data collection

We recorded the clinical data of all participants from the electronic medical record system of hospital, including demographics (gender, age, medical history and outcome), clinical symptoms, vital signs, and laboratory data (white blood cell [WBC], neutrophils, lymphocytes, monocytes, hemoglobin [HGB], platelet [PLT], alanine aminotransaminase [ALT], aspartate aminotransferase [AST], albumin [ALB], creatine phosphokinase [CK], lactate dehydrogenase [LDH], urea, creatinine [CREA], prothrombin time [PT], activated partial thromboplastin time [APTT], fibrinogen [FIB], high-sensitivity C-reactive protein [Hs-CRP], procalcitonin [PCT], IL-6, interleukin-10 [IL-10], interferon-gamma [IFN-γ], tumor necrosis factor-alpha [TNF-α]: IL-6/LY, neutrophil to lymphocyte ratio [NLR], platelet-to-lymphocyte ratio [PLR]). All data were dual-checked by two trained researchers.

### 2.3 Definitions and endpoint

Hemorrhage was defined as the presence of one or more of the following manifestations: oral or gingival bleeding, cutaneous ecchymosis or petechiae, hematuria, hemoptysis, hematemesis, melena, or hematochezia. Neurological signs were defined as the presence of at least one of the following abnormalities: altered consciousness, abnormal muscle tone, involuntary movements, or abnormal neurological reflexes. The study endpoint was patient outcome, categorized as death or survival. Death was defined as all-cause mortality. Survivors were defined as patients who met the discharge criteria, with normalization of body temperature and resolution of clinical symptoms. Patients who discontinued treatment or were discharged prematurely for personal reasons were followed up for outcome assessment until 28 days after symptom onset.

### 2.4 Ethics statement

This research was conducted in conformity with the Helsinki Declaration and was approved by the Local Ethics Committee of the Leading Center of Beijing Ditan Hospital, Capital Medical University (No. DTEC-KY2022-022-03). Written informed consent was obtained from all subjects.

### 2.5 Statistical analysis

Normally distributed data were presented as mean ± standard deviation (SD), and were compared between groups using the independent-samples t-test. Non-normally distributed measurements were reported as median with interquartile range (IQR) and were compared between groups using the Mann-Whitney U test. Categorical variables were expressed as n (%) and were analyzed by the chi-square test or the Fisher’s exact test. To mitigate the effect of extreme values and achieve approximate normality, cytokine concentrations and IL-6/LY were log10 transformed prior to statistical analysis. Univariate logistic regression was first performed to identify potential prognostic factors. Candidate predictors were selected based on prior literature and the univariate logistic regression results (*P* < 0.05). After excluding multicollinearity using the variance inflation factor (VIF), the remaining variables were included in the final multivariable model. The predictive ability of each indicator for mortality was evaluated using receiver operating characteristic (ROC) curve analysis, with the optimal cutoff value determined by Youden’s index. Differences between the AUCs of the biomarkers were compared using DeLong’s test. To assess the incremental prognostic value of IL-6/LY beyond established risk factors, the net reclassification improvement (NRI) and integrated discrimination improvement (IDI) were calculated. Cumulative survival rates between groups stratified by high and low IL-6/LY levels were compared using the Kaplan-Meier method, and differences were assessed with the log-rank test. The correlation between IL-6/LY and various clinical parameters was examined using Spearman’s correlation analysis. Clinical benefit was assessed by decision curve analysis (DCA). A two-sided *P* value < 0.05 was considered statistically significant. All statistical analyses were performed using SPSS version 25.0 (IBM, Armonk, NY, USA), R Studio version 4.5.2 and GraphPad Prism 8.0.

## 3 Results

### 3.1 Demographics and clinical characteristics

**Figure 1:**
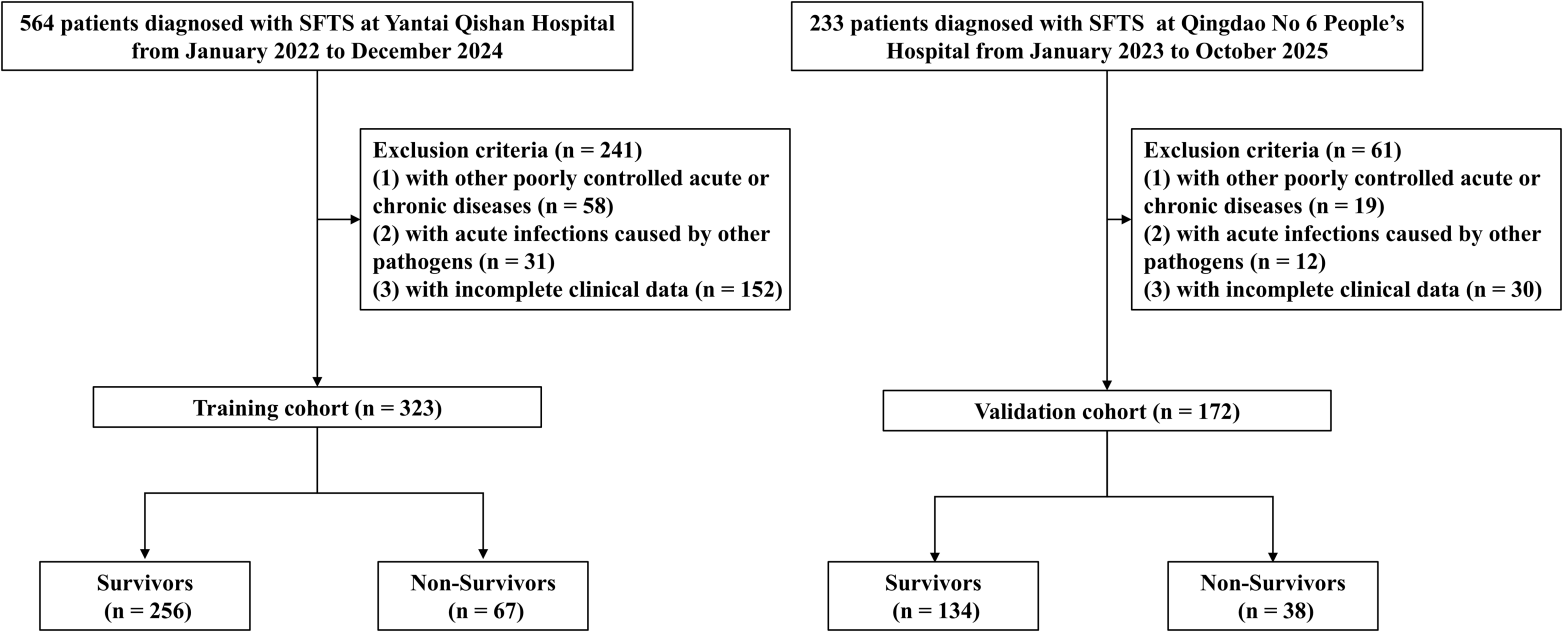
Flow diagram of patient selection for the training and external validation cohorts. SFTS, severe fever with thrombocytopenia syndrome.

A total of 495 patients diagnosed with SFTS were finally enrolled, including 323 patients hospitalized at Yantai Qishan Hospital from January 2022 to December 2024 in the training cohort, and 172 patients hospitalized at Qingdao No 6 People’s Hospital from January 2023 to October 2025 in the validation cohort (**Figure 1**). The median age of all cohorts at admission was 67 years (IQR, 60–74 years), and 218 (44.04%) patients were male (**Supplementary Table 1**). The training cohort comprised 323 patients with SFTS, including 256 (79.26%) survivors and 67 (20.74%) non−survivors. The median age was 67 years, with non−survivors being older than survivors (71 *vs*. 66 years). The study population included 149 males (46.1%), with no significant difference in sex distribution between the survivor and non−survivor groups. Compared with survivors, non−survivors showed a higher incidence of chest distress, hemorrhage, and neurological signs (all *P* < 0.05). Non-survivors also had significantly higher levels of DBV RNA, AST, CK, LDH, urea, CREA, PT, APTT, Hs-CRP, PCT, IL-6, IL-10, IFN-γ, TNF-α and IL-6/LY compared with survivors (all *P* < 0.05). Conversely, levels of lymphocytes, monocytes, PLT, ALB, and FIB were significantly lower in the non-survivor group (all *P* < 0.05) (**Table 1**). Similar differences between non−survivors and survivors were observed in the validation cohort (**Supplementary Table 2**).

**Table 1:** Clinical and laboratory characteristics of survivors and non-survivors in the training cohort of SFTS patients.

Characteristics	Total (n = 323)	Survivors (n = 256)	Non-Survivors (n = 67)	*P* value
Age, year	67.00 (59.00, 74.00)	66.00 (59.00, 72.00)	71.00 (64.00, 77.00)	**< 0.001**
Gender, (male), n (%)	149 (46.13)	120 (46.88)	29 (43.28)	0.600
Days from symptom onset	5.00 (4.00, 7.00)	5.00 (4.00, 7.00)	5.00 (4.00, 7.00)	0.191
Comorbidities, n (%)				
Diabetes	52 (16.10)	38 (14.84)	14 (20.90)	0.230
Hypertension	88 (27.24)	69 (26.95)	19 (28.36)	0.818
Cardiovascular disease	11 (3.41)	9 (3.52)	2 (2.99)	0.831
Cerebrovascular disease	16 (4.95)	14 (5.47)	2 (2.99)	0.404
Symptoms, n (%)				
Fever	306 (94.74)	242 (94.53)	64 (95.52)	0.746
Shiver	53 (16.41)	40 (15.63)	13 (19.40)	0.457
Fatigue	274 (84.83)	217 (84.77)	57 (85.07)	0.950
Inappetence	267 (82.66)	214 (83.59)	53 (79.10)	0.388
Nausea	155 (47.99)	127 (49.61)	28 (41.79)	0.254
Vomit	91 (28.17)	73 (28.52)	18 (26.87)	0.789
Abdominal pain	31 (9.60)	27 (10.55)	4 (5.97)	0.258
Diarrhea	123 (38.08)	92 (35.94)	31 (46.27)	0.121
Chest distress	22 (6.81)	12 (4.69)	10 (14.93)	**0.003**
Palpitation	15 (4.64)	10 (3.91)	5 (7.46)	0.218
Myalgia	117 (36.22)	97 (37.89)	20 (29.85)	0.223
Signs, n (%)				
Hemorrhage	45 (13.93)	24 (9.38)	21 (31.34)	**< 0.001**
Lymphadenectasis	103 (31.89)	81 (31.64)	22 (32.84)	0.852
Neurological signs	86 (26.63)	60 (23.44)	26 (38.81)	**0.011**
Laboratory parameters				
DBV RNA (log_10_ TCID50/mL)	3.29 ± 1.53	2.92 ± 1.38	4.71 ± 1.23	**< 0.001**
WBC (10^9^/L)	2.16 (1.56, 3.49)	2.32 (1.57, 3.59)	1.95 (1.43, 2.81)	0.121
Neutrophils (10^9^/L)	1.30 (0.88, 2.10)	1.29 (0.84, 2.10)	1.35 (0.99, 2.33)	0.603
Lymphocytes (10^9^/L)	0.46 (0.32, 0.77)	0.52 (0.33, 0.83)	0.41 (0.27, 0.52)	**0.001**
Monocytes (10^9^/L)	0.15 (0.08, 0.35)	0.16 (0.09, 0.36)	0.10 (0.06, 0.28)	**0.003**
HGB (g/L)	144.00 (132.00, 154.00)	144.00 (132.00, 154.00)	142.00 (133.00, 154.00)	0.726
PLT (10^9^/L)	67.00 (49.00, 88.00)	69.00 (52.00, 94.00)	51.00 (39.00, 70.00)	**< 0.001**
ALT (U/L)	62.40 (39.10, 114.50)	59.85 (35.63, 114.30)	68.80 (50.90, 125.30)	0.110
AST (U/L)	128.00 (68.60, 268.10)	113.55 (64.30, 234.60)	204.40 (127.00, 443.90)	**< 0.001**
ALB (g/L)	32.59 ± 4.89	33.11 ± 4.75	30.60 ± 4.94	**< 0.001**
CK (U/L)	449.00 (191.00, 1133.00)	387.50 (174.25, 998.50)	770.00 (300.00, 1331.00)	**0.003**
LDH (U/L)	549.00 (374.00, 932.00)	494.00 (352.25, 801.50)	851.00 (572.00, 1600.00)	**< 0.001**
UREA (mmol/L)	5.95 (4.51, 8.37)	5.71 (4.28, 7.54)	8.27 (5.60, 11.56)	**< 0.001**
CREA (umol/L)	67.00 (53.30, 84.20)	65.90 (53.00, 79.98)	78.00 (59.00, 103.00)	**0.002**
PT (sec)	13.00 (12.50, 13.70)	12.90 (12.40, 13.50)	13.40 (12.80, 14.10)	**< 0.001**
APTT (sec)	48.80 (42.60, 56.20)	47.65 (41.90, 53.10)	56.20 (49.00, 67.20)	**< 0.001**
FIB (g/L)	2.56 (2.15, 2.93)	2.60 (2.25, 3.00)	2.32 (1.97, 2.78)	**0.001**
Hs-CRP (mg/L)	3.20 (1.09, 8.94)	2.65 (0.87, 7.72)	7.56 (2.56, 13.32)	**< 0.001**
PCT (ng/ml)	0.16 (0.09, 0.44)	0.13 (0.08, 0.29)	0.47 (0.16, 0.76)	**< 0.001**
IL-6 (log_10_ pg/ml)	1.37 ± 0.73	1.19 ± 0.65	2.04 ± 0.64	**< 0.001**
IL-10 (log_10_ pg/ml)	1.12 ± 0.67	0.96 ± 0.62	1.75 ± 0.49	**< 0.001**
IFN-γ (log_10_ pg/ml)	2.05 (1.27, 2.52)	1.88 (1.09, 2.40)	2.51 (2.11, 2.94)	**< 0.001**
TNF-α (log_10_ pg/ml)	0.23 (0.00, 0.34)	0.22 (0.00, 0.32)	0.28 (0.11, 0.40)	**0.026**
IL-6/LY (log_10_)	1.67 ± 0.84	1.46 ± 0.78	2.44 ± 0.61	**< 0.001**

SFTS, severe fever with thrombocytopenia syndrome; DBV, Bandavirus dabieense; WBC, white blood cell; HGB, hemoglobin; PLT, platelet; ALT, alanine aminotransaminase; AST, aspartate aminotransferase; ALB, albumin; CK, creatine phosphokinase; LDH, lactate dehydrogenase; CREA, creatinine; PT, prothrombin time; APTT, activated partial thromboplastin time; FIB, fibrinogen; Hs-CRP, high-sensitivity C-reactive protein; PCT, procalcitonin; IL-6, interleukin-6; IL-10, interleukin-10; IFN-γ, interferon-gamma; TNF-α, tumor necrosis factor-alpha; IL-6/LY, interleukin-6-to-lymphocyte ratio. Continuous variable data are presented as mean (SD), median (interquartile ranges, IQR). Classified variable data are presented as n (%). P values indicate differences between survivors and non-survivors in the training cohort. *P* < 0.05 was considered statistically significant. Statistically significant values are shown in bold.

### 3.2 Independent risk factors for non-survivors in the training cohort

Univariate logistic regression identified age, DBV RNA, lymphocytes, PLT, AST, ALB, LDH, urea, PT, APTT, IL−6, IL−10, IFN−γ, TNF−α, and IL−6/LY as significant predictors of mortality (all *P* < 0.05). Based on previous studies and the univariate logistic regression results, seven variables—age, DBV RNA, PLT, urea, AST, PT, and IL-6/LY—were selected as candidate risk factors. After excluding variables with multicollinearity (VIF ≥ 2) (**Supplementary Table 3**), these seven variables were included in the multivariable logistic regression model. Multivariable logical regression model revealed that age (OR = 1.049, 95% CI: 1.009–1.091; *P* = 0.016), DBV RNA (OR = 2.453, 95% CI: 1.645–3.657; *P* < 0.001), and IL−6/LY (OR = 3.060, 95% CI: 1.724–5.432; *P* < 0.001) were independent risk factors for mortality (**Table 2**).

**Table 2:** Univariate and multivariate analysis of risk factors related to the mortality of SFTS patients in the training cohort.

Variables	Univariate analysis		Multivariate analysis	
	OR (95% CI)	*P* value	OR (95% CI)	*P* value
Age	1.058 (1.026-1.090)	**< 0.001**	1.049 (1.009-1.091)	**0.016**
Gender	0.865 (0.503-1.487)	0.600		
DBV RNA	3.049 (2.241-4.148)	**< 0.001**	2.453 (1.645-3.657)	**< 0.001**
WBC	0.900 (0.773-1.048)	0.174		
Neutrophils	0.934 (0.794-1.098)	0.407		
Lymphocytes	0.246 (0.096-0.628)	**0.003**		
Monocytes	0.976 (0.517-1.842)	0.941		
HGB	0.998 (0.983-1.014)	0.841		
PLT	0.975 (0.963-0.986)	**< 0.001**		
ALT	1.001 (0.999-1.003)	0.334		
AST	1.001 (1.001-1.002)	**0.001**		
ALB	0.902 (0.853-0.954)	**< 0.001**		
CK	1.000 (1.000-1.000)	0.207		
LDH	1.001 (1.001-1.001)	**< 0.001**		
UREA	1.141 (1.073-1.213)	**< 0.001**		
CREA	1.000 (0.999-1.001)	0.960		
PT	1.325 (1.069-1.642)	**0.010**		
APTT	1.051 (1.030-1.073)	**< 0.001**		
FIB	0.910 (0.689-1.201)	0.504		
Hs-CRP	1.012 (1.000-1.023)	0.055		
PCT	1.026 (0.993-1.059)	0.120		
IL-6	6.988 (4.105-11.897)	**< 0.001**		
IL-10	11.136 (5.806-21.261)	**< 0.001**		
IFN-γ	3.813 (2.386-6.093)	**< 0.001**		
TNF-α	3.332 (1.374-8.081)	**0.008**		
IL-6/LY	6.333 (3.856-10.402)	**< 0.001**	3.060 (1.724-5.432)	**< 0.001**

SFTS, severe fever with thrombocytopenia syndrome; DBV, Bandavirus dabieense; 95% CI, 95% confidence interval; OR, odds ratio; WBC, white blood cell; HGB, hemoglobin; PLT, platelet; ALT, alanine aminotransaminase; AST, aspartate aminotransferase; ALB, albumin; CK, creatine phosphokinase; LDH, lactate dehydrogenase; CREA, creatinine; PT, prothrombin time; APTT, activated partial thromboplastin time; FIB, fibrinogen; Hs-CRP, high-sensitivity C-reactive protein; PCT, procalcitonin; IL-6, interleukin-6; IL-10, interleukin-10; IFN-γ, interferon-gamma; TNF-α, tumor necrosis factor-alpha; IL-6/LY, interleukin-6-to-lymphocyte ratio. *P* < 0.05 was considered statistically significant. Statistically significant values are shown in bold.

### 3.3 Prognostic value and clinical benefit of IL-6/LY for mortality in patients with SFTS

**Figure 2:**
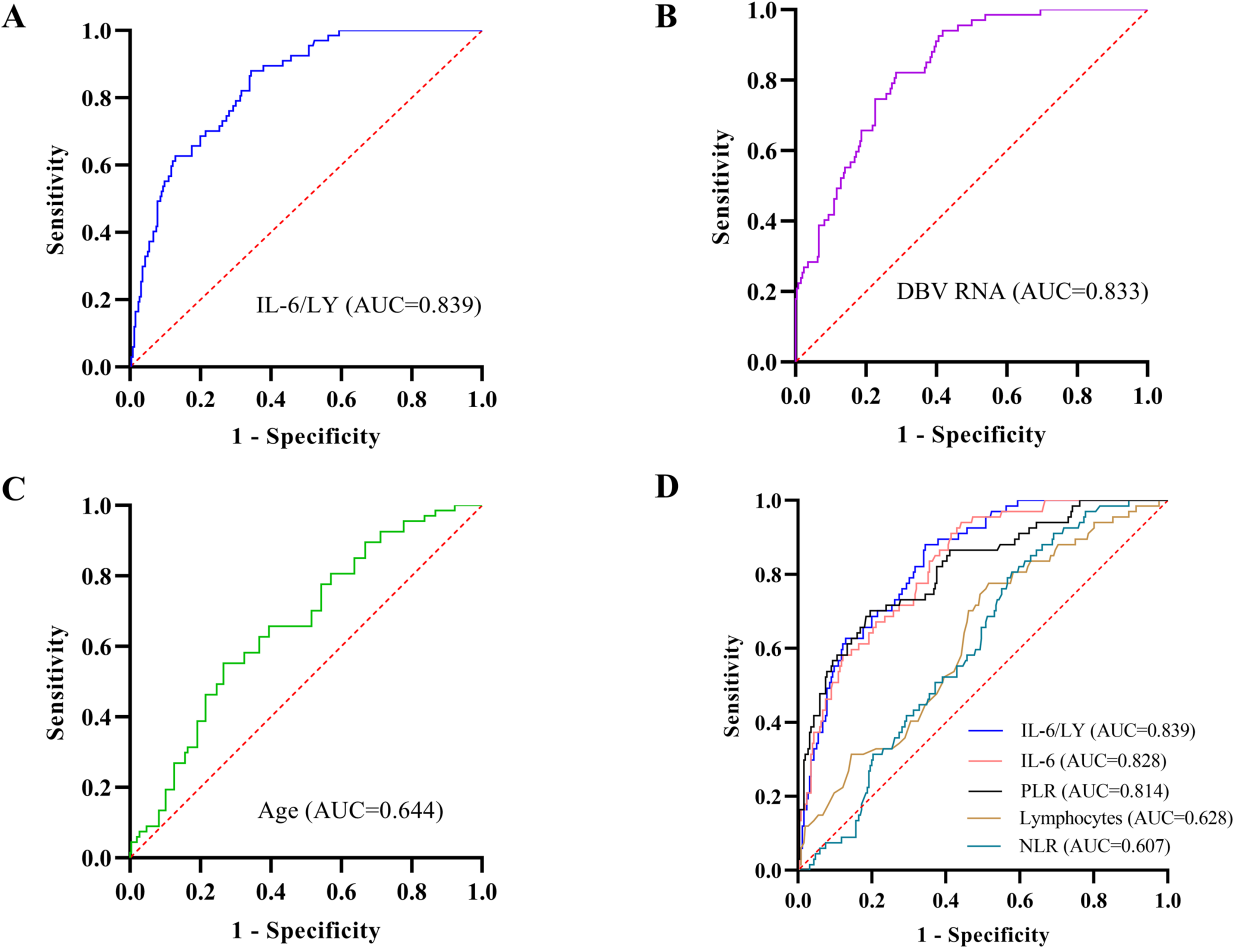
ROC curves analysis of prognostic indicators for mortality in patients with SFTS in the training cohort. **(A)** IL-6/LY had an AUC of 0.839 (95% CI: 0.792–0.886, *P* < 0.001). **(B)** DBV RNA had an AUC of 0.833 (95%CI: 0.786–0.881, *P* < 0.001). **(C)** Age had an AUC of 0.644 (95% CI: 0.574–0.715, *P* < 0.001). **(D)** IL-6/LY had an AUC of 0.839 (95% CI: 0.792–0.886, *P* < 0.001); IL-6 had an AUC of 0.828 (95% CI: 0.778–0.878, *P* < 0.001); PLR had an AUC of 0.814 (95% CI: 0.755–0.873, *P* < 0.001); Lymphocytes had an AUC of 0.628 (95% CI: 0.555–0.700, *P* = 0.001); NLR had an AUC of 0.607 (95% CI: 0.540–0.674, *P* = 0.007). ROC, receiver operating characteristic; SFTS, severe fever with thrombocytopenia syndrome; DBV, Bandavirus dabieense; AUC, area under the curve; 95% CI, 95% confidence interval; IL-6/LY, interleukin-6-to-lymphocyte ratio; IL-6, interleukin-6; PLR, platelet-to-lymphocyte ratio; NLR, neutrophil-to-lymphocyte ratio.

To evaluate the prognostic performance of IL−6/LY for fatal outcomes in patients with SFTS, its predictive ability was compared with that of the other independent risk factors identified above. As shown in **Figure 2** and **Table 3**, the area under the ROC curve (AUC) for IL−6/LY was 0.839 (95% CI: 0.792-0.886, *P* < 0.001) (**Figure 2A**), whereas the AUCs for DBV RNA and age were 0.833 (95% CI: 0.786-0.881, *P* < 0.001) and 0.644 (95% CI: 0.574-0.715, *P* < 0.001), respectively (**Figures 2B, C**). Compared with NLR and PLR, IL−6/LY exhibited superior discriminatory power for predicting mortality (**Figure 2D**). Comparison of ROC curves using DeLong’s test revealed that the AUC of IL-6/LY was significantly higher than that of lymphocytes in both the training (*P* < 0.001) and validation cohorts (*P* < 0.001). Compared to IL-6 alone, the AUC of IL-6/LY was numerically higher but the difference was not statistically significant in the training (0.839 *vs*. 0.828, *P* = 0.282) or validation cohorts (0.812 *vs*. 0.805, *P* = 0.701) (**Supplementary Tables 4**, **5**). The optimal cutoff value for IL−6/LY, determined by the maximum Youden index, was 1.77, yielding a sensitivity of 88.10% and a specificity of 64.80% (**Table 3**).

**Table 3:** Predictive value of prognostic indicators for mortality in patients with SFTS in the training cohort.

Parameters	AUC	Cutoff value	Sensitivity (%)	Specificity (%)	95% CI	*P* value
Age	0.644	70.50	55.20%	70.30%	0.574-0.715	**< 0.001**
DBV RNA	0.833	3.77	82.10%	70.70%	0.786-0.881	**< 0.001**
IL-6/LY	0.839	1.77	88.10%	64.80%	0.792-0.886	**< 0.001**
Lymphocytes	0.628	0.54	77.60%	48.40%	0.555-0.700	**0.001**
IL-6	0.828	1.27	94.00%	55.90%	0.778-0.878	**< 0.001**
NLR	0.607	2.20	80.60%	42.20%	0.540-0.674	**0.007**
PLR	0.814	96.30	70.10%	80.50%	0.755-0.873	**< 0.001**

SFTS, severe fever with thrombocytopenia syndrome; DBV, Bandavirus dabieense; AUC, area under the curve; 95% CI, 95% confidence interval; IL-6, interleukin-6; IL-6/LY, interleukin-6-to-lymphocyte ratio; NLR, neutrophil-to-lymphocyte ratio; PLR, platelet-to-lymphocyte ratio. *P* < 0.05 was considered statistically significant. Statistically significant values are shown in bold.

To further assess the incremental prognostic value of IL-6/LY beyond the two confirmed independent risk factors (age and DBV RNA) identified by our previous multivariable logistic regression analysis, we constructed two nested multivariable logistic regression models and quantified the model performance improvement using NRI and IDI. Model 1 included age and DBV RNA, and Model 2 added IL-6/LY to Model 1. Analyses were performed in the training, validation, and combined cohorts to ensure the robustness of the results. Incorporation of IL−6/LY significantly improved risk prediction in the training cohort, with a continuous NRI of 0.676 (95% CI: 0.419–0.933, *P* < 0.001) and an IDI of 0.063 (95% CI: 0.029–0.097, *P* < 0.001). Similar significant improvements were observed in the validation cohort (continuous NRI: 0.795, 95% CI: 0.479–1.111, *P* < 0.001; IDI: 0.075, 95% CI: 0.022–0.129, *P* = 0.006). Notably, these findings were further validated in the combined cohort, with a continuous NRI of 0.732 (95% CI: 0.531–0.933, *P* < 0.001) and an IDI of 0.066 (95% CI: 0.038–0.094, *P* < 0.001), confirming the robust incremental prognostic value of IL-6/LY independent of age and DBV RNA (**Supplementary Table 6**).

**Figure 3:**
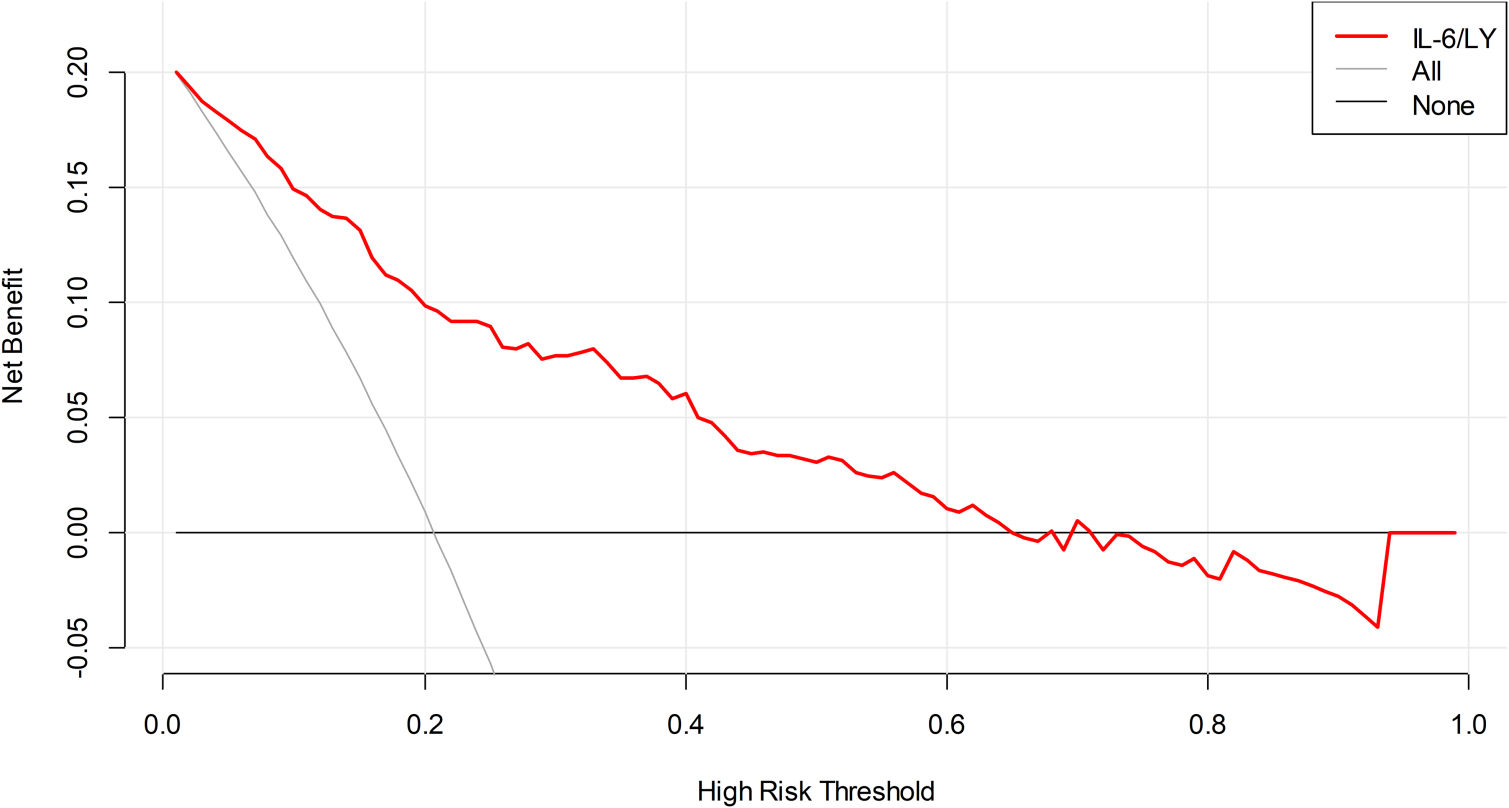
DCA of IL-6/LY for mortality in patients with SFTS in the training cohort. DCA, decision curve analysis; IL-6/LY, interleukin-6 to lymphocyte ratio; SFTS, severe fever with thrombocytopenia syndrome.

Decision curve analysis demonstrated that IL−6/LY yielded a greater net benefit than either the treat−all or treat−none strategy across a wide range of threshold probabilities, indicating its superior clinical utility (**Figure 3**).

### 3.4 Clinical characteristics of SFTS patients stratified by IL-6/LY levels

IL−6/LY was identified as an independent risk factor for mortality in patients with SFTS. All patients were divided into high IL−6/LY and low IL−6/LY groups according to the cutoff value of 1.77. Patients in the high IL−6/LY group were significantly older than those in the low IL−6/LY group (P = 0.001). Compared with the low IL−6/LY group, the high IL−6/LY group had a significantly higher mortality rate (P < 0.001) and increased incidence of myalgia (P = 0.005), hemorrhage (P = 0.008), and neurological manifestations (P = 0.035). Moreover, patients in the high IL-6/LY group demonstrated higher levels of DBV RNA, AST, CK, LDH, urea, CREA, PT, APTT, Hs-CRP, PCT, IL-6, IL-10, IFN-γ, and TNF-α than those in the low IL−6/LY group (all P < 0.05). In contrast, levels of WBC, lymphocytes, monocytes, PLT, ALB, and FIB were significantly lower in the high IL-6/LY group (all P < 0.001) (**Table 4**).

**Table 4:** Clinical and laboratory characteristics of patients with SFTS stratified by the IL-6/LY cutoff value on admission in the training cohort.

Characteristics	Total (n = 323)	IL-6/LY ^low^ (< 1.77) (n = 174)	IL-6/LY ^high^ (≥ 1.77) (n = 149)	*P* value
Age, year	67.00 (59.00, 74.00)	65.00 (58.00, 71.00)	68.00 (62.00, 76.00)	**0.001**
Gender, (male), n (%)	149 (46.13)	74 (49.66)	75 (50.34)	0.238
Outcome, (death), n (%)	67 (20.74)	8 (4.60)	59 (39.60)	**< 0.001**
Comorbidities, n (%)				
Diabetes	52 (16.10)	27 (15.52)	25 (16.78)	0.758
Hypertension	88 (27.24)	49 (28.16)	39 (26.17)	0.689
Cardiovascular disease	11 (3.41)	6 (3.45)	5 (3.36)	0.964
Cerebrovascular disease	16 (4.95)	9 (5.17)	7 (4.70)	0.845
Symptoms, n (%)				
Fever	306 (94.74)	166 (95.40)	140 (93.96)	0.563
Shiver	53 (16.41)	25 (14.37)	28 (18.79)	0.285
Fatigue	274 (84.83)	145 (83.33)	129 (86.58)	0.418
Inappetence	267 (82.66)	144 (82.96)	123 (82.55)	0.961
Nausea	155 (47.99)	90 (51.72)	65 (43.62)	0.146
Vomit	91 (28.17)	53 (30.46)	38 (25.50)	0.324
Abdominal pain	31 (9.60)	19 (10.92)	12 (8.05)	0.383
Diarrhea	123 (38.08)	62 (35.63)	61 (40.94)	0.327
Chest distress	22 (6.81)	8 (4.60)	14 (9.40)	0.088
Palpitation	15 (4.64)	6 (3.45)	9 (6.04)	0.270
Myalgia	117 (36.22)	75 (43.10)	42 (28.19)	**0.005**
Signs, n (%)				
Hemorrhage	45 (13.93)	16 (9.20)	29 (19.46)	**0.008**
Lymphadenectasis	103 (31.89)	59 (33.91)	44 (29.54)	0.400
Neurological signs	86 (26.63)	38 (21.84)	48 (32.21)	**0.035**
Laboratory parameters				
DBV RNA (log10 TCID50/mL)	3.29 ± 1.53	2.61 ± 1.38	4.10 ± 1.30	**< 0.001**
WBC (10^9^/L)	2.16 (1.56, 3.49)	2.52 (1.75, 3.71)	1.92 (1.37, 3.03)	**< 0.001**
Neutrophils (10^9^/L)	1.30 (0.88, 2.10)	1.29 (0.84, 2.11)	1.35 (0.91, 2.19)	0.717
Lymphocytes (10^9^/L)	0.46 (0.32, 0.77)	0.65 (0.43, 0.98)	0.36 (0.27, 0.51)	**< 0.001**
Monocytes (10^9^/L)	0.15 (0.08, 0.35)	0.23 (0.12, 0.42)	0.09 (0.06, 0.22)	**< 0.001**
HGB (g/L)	144.00 (132.00, 154.00)	143.50 (131.00, 156.00)	144.00 (132.50, 153.00)	0.720
PLT (10^9^/L)	67.00 (49.00, 88.00)	72.00 (54.00, 98.00)	58.00 (44.00, 77.00)	**< 0.001**
ALT (U/L)	62.40 (39.10, 114.50)	59.85 (38.63, 112.13)	66.80 (41.50, 128.40)	0.321
AST (U/L)	128.00 (68.60, 268.10)	103.50 (59.58, 223.05)	162.70 (92.75, 308.55)	**< 0.001**
ALB (g/L)	32.59 ± 4.89	33.62 ± 4.73	31.38 ± 4.81	**< 0.001**
CK (U/L)	449.00 (191.00, 1133.00)	382.50 (161.50, 1055.25)	552.00 (227.50, 1233.50)	**0.026**
LDH (U/L)	549.00 (374.00, 932.00)	462.50 (347.00, 728.00)	685.00 (441.50, 1147.00)	**< 0.001**
UREA (mmol/L)	5.95 (4.51, 8.37)	5.40 (4.03, 7.26)	6.88 (5.29, 9.86)	**< 0.001**
CREA (umol/L)	67.00 (53.30, 84.20)	62.65 (50.70, 75.25)	75.00 (59.50, 94.00)	**< 0.001**
PT (sec)	13.00 (12.50, 13.70)	12.80 (12.38, 13.33)	13.20 (12.80, 13.90)	**< 0.001**
APTT (sec)	48.80 (42.60, 56.20)	46.15 (39.50, 51.23)	52.10 (47.15, 63.05)	**< 0.001**
FIB (g/L)	2.56 (2.15, 2.93)	2.69 (2.28, 3.06)	2.39 (2.04, 2.81)	**< 0.001**
Hs-CRP (mg/L)	3.20 (1.09, 8.94)	1.95 (0.52, 5.35)	5.62 (2.57, 13.01)	**< 0.001**
PCT (ng/ml)	0.16 (0.09, 0.44)	0.11 (0.07, 0.22)	0.28 (0.12, 0.71)	**< 0.001**
IL-6 (log_10_ pg/ml)	1.36 ± 0.73	0.86 ± 0.45	1.96 ± 0.53	**< 0.001**
IL-10 (log_10_ pg/ml)	1.12 ± 0.67	0.81 ± 0.61	1.50 ± 0.54	**< 0.001**
IFN-γ (log_10_ pg/ml)	2.05 (1.27, 2.52)	1.62 (0.89, 2.10)	2.49 (2.09, 2.83)	**< 0.001**
TNF-α (log_10_ pg/ml)	0.23 (0.00, 0.34)	0.18 (-0.04, 0.32)	0.32 (0.10, 0.38)	**< 0.001**

SFTS, severe fever with thrombocytopenia syndrome; DBV, Bandavirus dabieense; WBC, white blood cell; HGB, hemoglobin; PLT, platelet; ALT, alanine aminotransaminase; AST, aspartate aminotransferase; ALB, albumin; CK, creatine phosphokinase; LDH, lactate dehydrogenase; CREA, creatinine; PT, prothrombin time; APTT, activated partial thromboplastin time; FIB, fibrinogen; Hs-CRP, high-sensitivity C-reactive protein; PCT, procalcitonin; IL-6, interleukin-6; IL-10, interleukin-10; IFN-γ, interferon-gamma; TNF-α, tumor necrosis factor-alpha; IL-6/LY, interleukin-6-to-lymphocyte ratio. Continuous variable data are presented as mean (SD), median (interquartile ranges, IQR). Classified variable data are presented as n (%). *P* < 0.05 was considered statistically significant. Statistically significant values are shown in bold.

**Figure 4:**
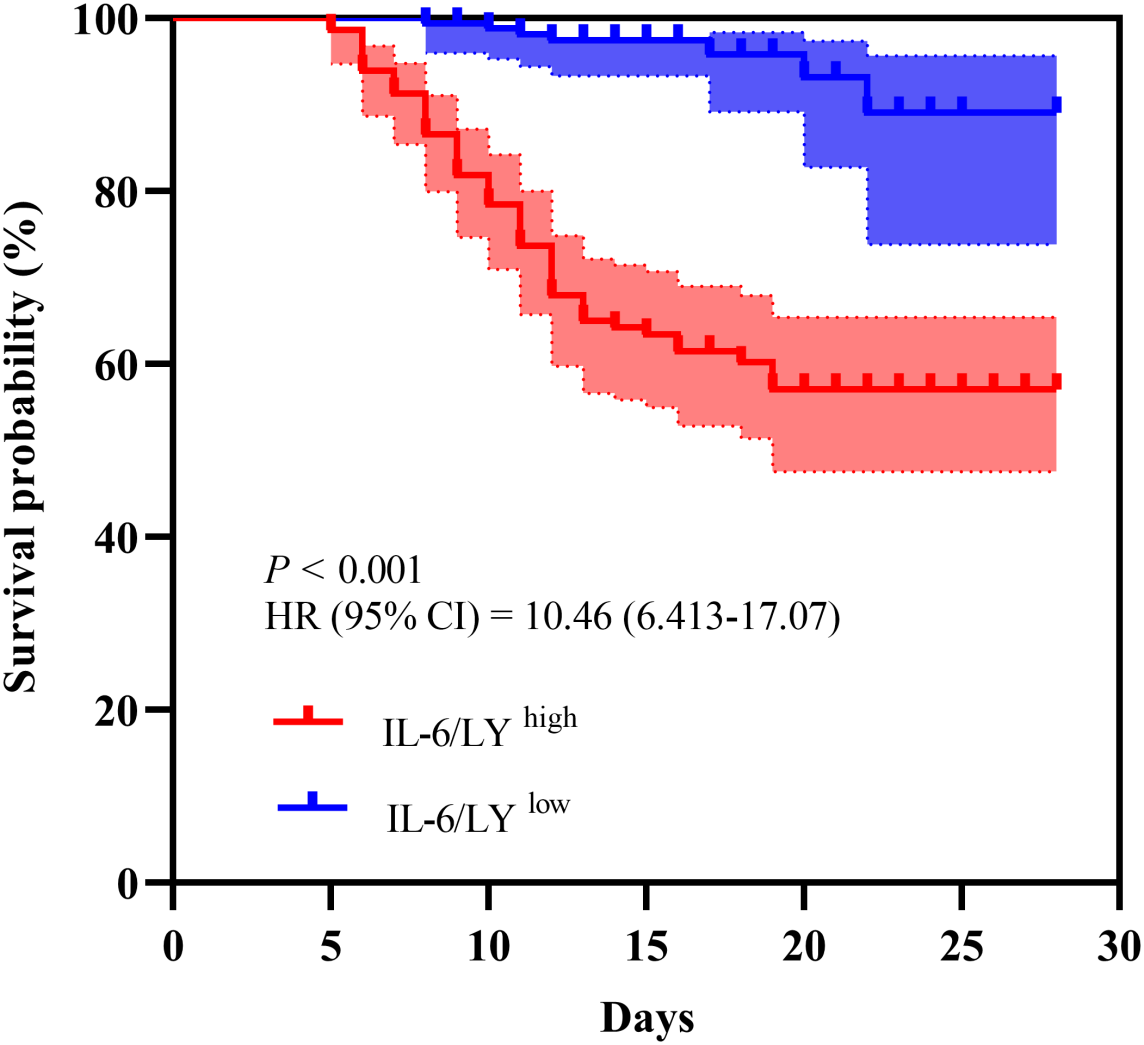
Kaplan-Meier survival curves comparing mortality in SFTS patients based on the cutoff value of IL6/LY in the training cohort. SFTS, severe fever with thrombocytopenia syndrome; IL-6/LY, interleukin-6-to-lymphocyte ratio, HR, hazard ratio.

A Kaplan–Meier survival analysis was performed to assess the prognostic value of IL−6/LY within 28 days after disease onset in patients with SFTS. As shown in **Figure 4**, Patients with high IL−6/LY levels showed significantly poorer survival than those with low IL−6/LY (log−rank *P* < 0.001). This finding further supports the association between elevated IL−6/LY levels and adverse outcomes in patients with SFTS.

### 3.5 Correlations between IL-6/LY and laboratory parameters

Spearman’s rank correlation analysis revealed that IL-6/LY was positively correlated with DBV RNA (r = 0.599, *P* < 0.001), AST (r = 0.318, *P* < 0.001), CK (r = 0.220, *P* < 0.001), LDH (r = 0.357, *P* < 0.001), urea (r = 0.354, *P* < 0.001), CREA (r = 0.361, *P* < 0.001), PT (r = 0.311, *P* < 0.001), and APTT (r = 0.462, *P* < 0.001), whereas it was negatively correlated with ALB (r = -0.340, *P* < 0.001) and FIB (r = -0.223, *P* < 0.001) (**Table 5**).

**Table 5:** Correlation between IL-6/LY and laboratory parameters in SFTS patients in the training cohort.

Parameters	r	*P* value
DBV RNA	0.599	**<0.001**
AST	0.318	**<0.001**
ALB	-0.340	**<0.001**
CK	0.220	**<0.001**
LDH	0.357	**<0.001**
UREA	0.354	**<0.001**
CREA	0.361	**<0.001**
PT	0.311	**<0.001**
APTT	0.462	**<0.001**
FIB	-0.223	**<0.001**

IL-6/LY, interleukin-6 to lymphocyte ratio; SFTS, severe fever with thrombocytopenia syndrome, AST, aspartate aminotransferase; ALB, albumin; CK, creatine phosphokinase; LDH, lactate dehydrogenase; CREA, creatinine; PT, prothrombin time; APTT, activated partial thromboplastin time.

*P* < 0.05 was considered statistically significant. Statistically significant values are shown in bold.

### 3.6 External validation of IL-6/LY for mortality in patients with SFTS

**Figure 5:**
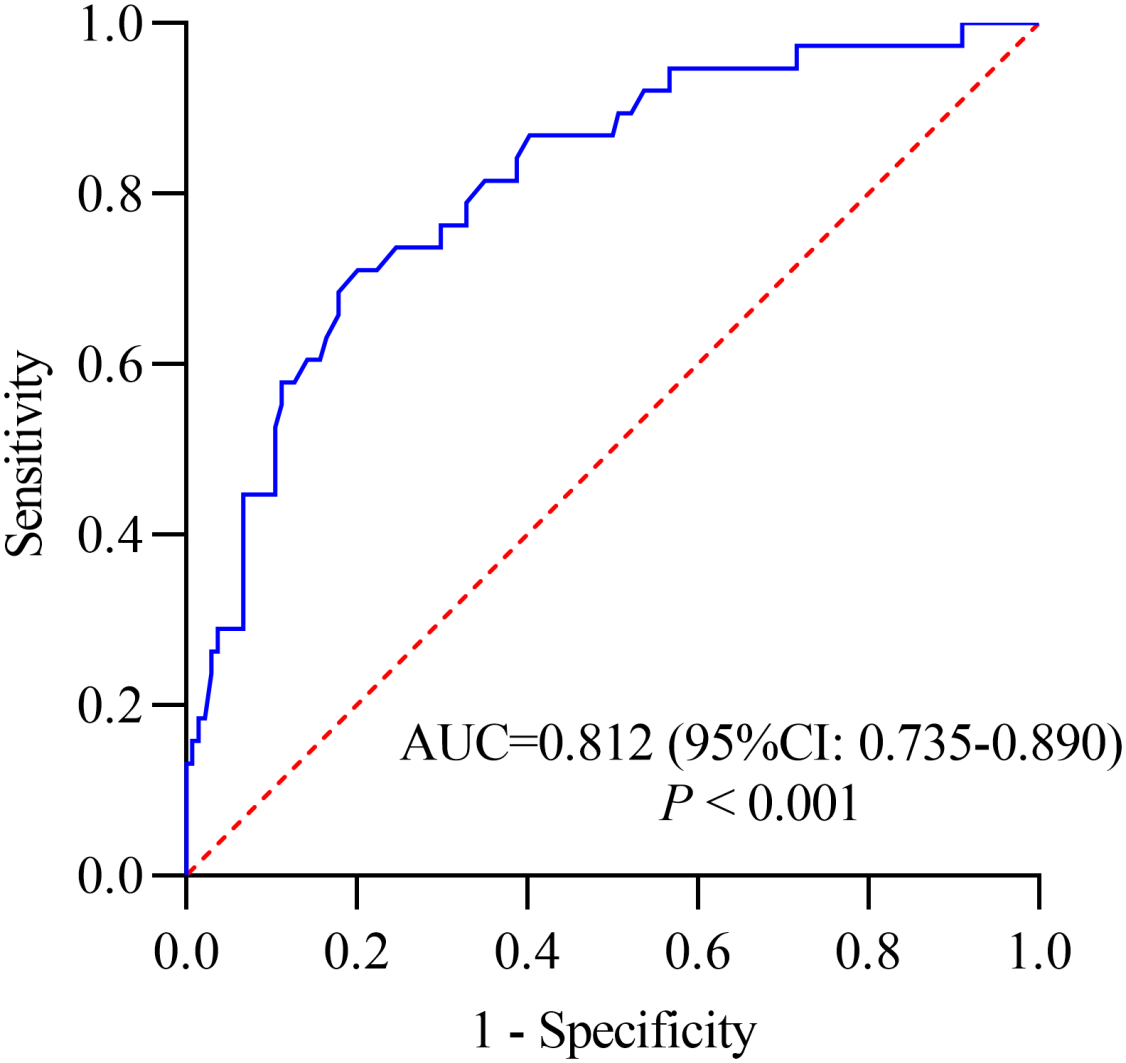
ROC curve analysis of IL-6/LY for mortality in patients with SFTS in the validation cohort. ROC, receiver operating characteristic; SFTS, severe fever with thrombocytopenia syndrome; 95% CI, 95% confidence interval; IL-6/LY, interleukin-6-to-lymphocyte ratio.

**Figure 6:**
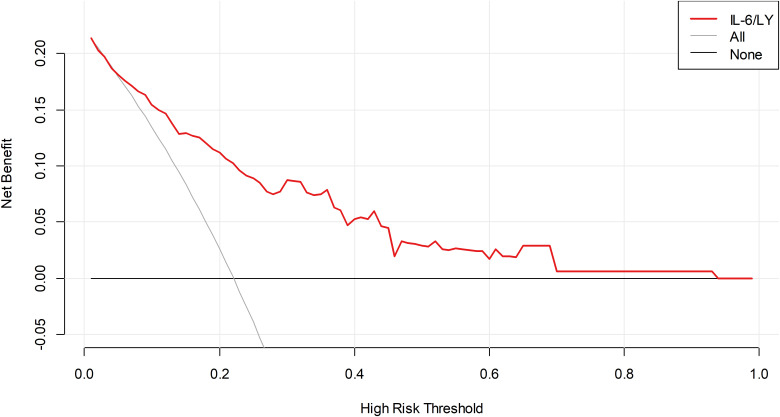
DCA of IL-6/LY for mortality in patients with SFTS in the validation cohort. DCA, decision curve analysis; IL-6/LY, interleukin-6-to-lymphocyte ratio; SFTS, severe fever with thrombocytopenia syndrome.

In the external validation cohort, IL−6/LY also demonstrated excellent discriminatory performance, with an AUC of 0.812 (95% CI: 0.735–0.890, *P* < 0.001) (**Figure 5**). Decision curve analysis further showed that IL−6/LY provided a substantial net clinical benefit across a wide range of threshold probabilities (**Figure 6**).

## 4 Discussion

This multicenter study is the first to evaluate the association between IL−6/LY and mortality in patients with SFTS. Our findings demonstrate that IL-6/LY is an independent predictor of mortality and shows excellent discriminative performance and clinical benefit across two independent cohorts.

Cytokine storm is a critical contributor to disease progression, leading to multiple organ failure and death (17–19). IL-6, a key proinflammatory cytokine induced by infection or tissue injury, is markedly elevated during cytokine storms and serves as one of their characteristic hallmarks (20, 21). IL−6 can bind to either the membrane−bound receptor (mIL−6R) or the soluble receptor (sIL−6R) in complex with the signal−transducing glycoprotein gp130, thereby activating the JAK/STAT3 and PI3K/AKT signaling pathways (22, 23). This activation induces the expression and release of proinflammatory chemokines and cytokines, including MCP-1, IL-8, and IL-10, in human vascular endothelial cells (24). Excessive IL-6 production has been strongly associated with disease severity and fatal outcomes in SFTS (25). Previous studies by Yoo et al. (26) and Liu et al. (27) reported significantly elevated IL-6 levels in fatal cases of SFTS compared with non-fatal cases. In line with these findings, our analysis similarly demonstrated that non-surviving SFTS patients exhibited substantially higher IL-6 levels than survivors, reinforcing the association between elevated IL-6 and fatal outcomes. These observations suggest that patients with markedly increased IL−6/LY may benefit from early initiation of immunomodulatory therapy (e.g., the IL−6 receptor antagonist tocilizumab). A recent prospective, longitudinal study reported that tocilizumab treatment significantly reduced IL−6 levels and was associated with improved clinical outcomes in SFTS (28). However, further studies are needed to confirm these findings.

Lymphocytes play a critical role in coordinating antiviral immune responses. Several studies have demonstrated that patients with severe or critical illness during viral infections (e.g., SARS-CoV-2, influenza virus, or DBV) exhibit significantly lower lymphocyte counts than healthy, mildly affected, or recovered individuals (29–33). Consistent with these observations, markedly reduced lymphocyte counts at admission in fatal SFTS cases compared with survivors, suggesting a close association between the degree of lymphopenia and disease severity. DBV infection leads to depletion of multiple major lymphocyte subsets, including T, B, and NK cells (34–36), yet the underlying mechanisms remain multifactorial and incompletely understood. Pyroptosis, a highly pro-inflammatory form of programmed cell death, has been implicated as a potential contributor. Li et al. reported that DBV infection activates the NOD-like receptor protein 3 (NLRP3) inflammasome, thereby enhancing IL-1β and IL-18 secretion and triggering pyroptosis in human peripheral blood mononuclear cells (PBMCs) as well as in murine models (37). Additionally, DBV has been shown to induce autophagy, exploiting this pathway for viral assembly and egress, which further exacerbates lymphocyte depletion (38). Activated macrophages during DBV infection further amplify immune dysregulation by releasing high levels of cytokines and chemokines that promote lymphopenia and suppress adaptive immune responses (39, 40). Notably, IL-6 can directly impair hematopoietic stem cells (41) and activates STAT3 (42), thereby inhibiting lymphocyte proliferation and survival.

The pathogenic mechanism of SFTS was closely associated with dysregulated host immune responses and excessive inflammatory damage induced by DBV infection (43, 44). IL-6/LY, a novel immune−inflammatory biomarker, overcomes the inherent limitations of relying on isolated inflammatory or immune parameters. By integrating key components of both pathways, it offers a more stable and reliable indicator, thereby strengthening its utility in prognostic stratification. Yang et al. reported that IL-6/LY showed superior discriminatory power for mortality prediction compared with NLR, with an AUC of 0.919 versus 0.882 in COVID-19 (16). Similarly, our study demonstrated that the predictive efficacy of IL-6/LY (AUC = 0.839) was numerically comparable to that of DBV RNA (AUC = 0.833) and markedly outperformed that of NLR (AUC = 0.607). When evaluating the individual components, the discrimination of IL−6/LY did not differ significantly from IL−6 alone, but was significantly better than lymphocytes alone (*P* < 0.001). Although IL−6 alone performed similarly, IL−6/LY integrates a marker of inflammatory activation with an index of host immune status, and therefore may more comprehensively reflect the imbalance between hyperinflammation and immune suppression observed in severe disease. Multivariate logistic analyses further identified IL−6/LY as an independent predictor of adverse outcomes. DCA further demonstrated excellent clinical benefit across a range of threshold probabilities, supporting its value for risk stratification in clinical practice. To explore the clinical implications of IL-6/LY, patients were stratified into low and high groups based on an optimal cutoff value. Patients with elevated IL-6/LY levels were older and exhibited higher viral loads, increased proinflammatory cytokine responses, and a greater frequency of hemorrhagic manifestations, neurological complications, and multi-organ dysfunction. Survival analysis further demonstrated significantly poorer outcomes in patients with high IL-6/LY. In addition, IL-6/LY showed strong correlations with laboratory indicators of cardiac, hepatic, renal, and coagulation dysfunction, suggesting that elevated IL-6/LY reflects systemic immune dysregulation and widespread organ injury, which may contribute to disease severity and mortality in SFTS. Importantly, the good predictive performance and clinical benefit of IL-6/LY were further supported by external validation, strengthening the robustness of our results. Baseline IL−6/LY could support earlier identification of high−risk patients, which could inform closer monitoring, timely escalation of care (including early ICU referral), and consideration of immunomodulatory strategies in appropriate settings. However, these findings require validation in future studies.

We acknowledge several limitations in this study. First, the modest sample size—especially in the fatal subgroup—and the absence of longitudinal sampling limit both the generalizability of our findings and the assessment of IL-6/LY trajectories over the course of SFTS progression. Second, despite multivariable adjustment for key confounders (e.g., age, viral load, other blood parameters), residual confounding from unmeasured factors and potential recall bias regarding pre-hospital therapy cannot be entirely ruled out. Third, the optimal IL-6/LY cutoff identified in the training cohort requires external validation across different populations, settings, and assay platforms to ensure its applicability. Therefore, multicenter prospective studies and experimental investigations are needed to further validate the prognostic value of IL-6/LY and to explore its underlying mechanisms in disease pathogenesis.

## 5 Conclusion

This study is the first to demonstrate that IL-6/LY is independently associated with early mortality in patients with SFTS. It may aid in the early identification of high-risk patients and potentially serve as a target for future therapeutic interventions.

## Data Availability

The original contributions presented in the study are included in the article/**Supplementary Material**. Further inquiries can be directed to the corresponding authors.

## References

[1] Yu XJ Liang MF Zhang SY Liu Y Li JD Sun YL . Fever with thrombocytopenia associated with a novel bunyavirus in China. N Engl J Med. (2011) 364:1523–32. doi: 10.1056/nejmoa1010095. PMID: 21410387 PMC3113718

[2] Kim YR Yun Y Bae SG Park D Kim S Lee JM . Severe fever with thrombocytopenia syndrome virus infection, South Korea, 2010. Emerg Infect Dis. (2018) 24:2103–5. doi: 10.3201/eid2411.170756. PMID: 30334706 PMC6199997

[3] Rattanakomol P Khongwichit S Linsuwanon P Lee KH Vongpunsawad S Poovorawan Y . Severe fever with thrombocytopenia syndrome virus infection, Thailand, 2019-2020. Emerg Infect Dis. (2022) 28:2572–4. doi: 10.3201/eid2812.221183. PMID: 36418010 PMC9707585

[4] Tran XC Yun Y Van An L Kim SH Thao NTP Man PKC . Endemic severe fever with thrombocytopenia syndrome, Vietnam. Emerg Infect Dis. (2019) 25:1029–31. doi: 10.3201/eid2505.181463. PMID: 31002059 PMC6478219

[5] Win AM Nguyen YTH Kim Y Ha NY Kang JG Kim H . Genotypic heterogeneity of Orientia tsutsugamushi in scrub typhus patients and thrombocytopenia syndrome co-infection, Myanmar. Emerg Infect Dis. (2020) 26:1878–81. doi: 10.3201/eid2608.200135. PMID: 32687023 PMC7392420

[6] Jiang XL Zhang S Jiang M Bi ZQ Liang MF Ding SJ . A cluster of person-to-person transmission cases caused by SFTS virus in Penglai, China. Clin Microbiol Infect. (2015) 21:274–9. doi: 10.1016/j.cmi.2014.10.006. PMID: 25687766

[7] Kiyotoki S Kurotaka R Tokunaga Y Takahashi T Shimojima M Yoshikawa T . First case of nosocomial transmission of severe fever with thrombocytopenia syndrome in Japan. Int J Infect Dis. (2025) 12:108057. doi: 10.1016/j.ijid.2025.108057. PMID: 40946779

[8] Cui H Shen S Chen L Fan Z Wen Q Xing Y . Global epidemiology of severe fever with thrombocytopenia syndrome virus in human and animals: a systematic review and meta-analysis. Lancet Reg Health West Pac. (2024) 48:101133. doi: 10.1016/j.lanwpc.2024.101133. PMID: 39040038 PMC11261768

[9] Li H Lu QB Xing B Zhang SF Liu K Du J . Epidemiological and clinical features of laboratory-diagnosed severe fever with thrombocytopenia syndrome in China, 2011-17: a prospective observational study. Lancet Infect Dis. (2018) 18:1127–37. doi: 10.1007/978-94-017-9882-2_28. PMID: 30054190

[10] Wei Y Wang Z Kang L He L Sheng N Qin J . NLR, a convenient early-warning biomarker of fatal outcome in patients with severe fever with thrombocytopenia syndrome. Front Microbiol. (2022) 13:907888. doi: 10.3389/fmicb.2022.907888. PMID: 35814714 PMC9262381

[11] Wang X Lin L Zhao Z Zhou W Ge Z Shen Y . The predictive effect of the platelet-to-lymphocyte ratio (PLR) and the neutrophil-to-lymphocyte ratio (NLR) on the risk of death in patients with severe fever with thrombocytopenia syndrome (SFTS): a multi-center study in China. Ann Transl Med. (2021) 9:208. doi: 10.21037/atm-20-4736. PMID: 33708835 PMC7940944

[12] Yang X Yin H Xiao C Li R Liu Y . The prognostic significance of C-reactive protein to albumin ratio in patients with severe fever with thrombocytopenia syndrome. Front Med (Lausanne). (2022) 9:879982. doi: 10.3389/fmed.2022.879982. PMID: 35572999 PMC9099431

[13] Yang Z Wang L Hong B He Z Zhang Q Shen T . Inflammatory burden index as a predictor of in-hospital mortality in patients with severe fever with thrombocytopenia syndrome. J Med Virol. (2025) 97:e70225. doi: 10.1002/jmv.70225. PMID: 39936887

[14] Song L Zou W Wang G Qiu L Sai L . Cytokines and lymphocyte subsets are associated with disease severity of severe fever with thrombocytopenia syndrome. Virol J. (2024) 21:126. doi: 10.1186/s12985-024-02403-0. PMID: 38831352 PMC11149350

[15] Kwon JS Jin S Kim JY Ra SH Kim T Park SY . Viral and immunologic factors associated with fatal outcome of patients with severe fever with thrombocytopenia syndrome in Korea. Viruses. (2021) 13:2351. doi: 10.3390/v13122351. PMID: 34960620 PMC8703577

[16] Yang B Chang X Huang J Pan W Si Z Zhang C . The role of IL-6/lymphocyte ratio in the peripheral blood of severe patients with COVID-19. Int Immunopharmacol. (2021) 97:107569. doi: 10.1016/j.intimp.2021.107569. PMID: 33933851 PMC7953449

[17] Hojyo S Uchida M Tanaka K Hasebe R Tanaka Y Murakami M . How COVID-19 induces cytokine storm with high mortality. Inflammation Regener. (2020) 40:37. doi: 10.1186/s41232-020-00146-3. PMID: 33014208 PMC7527296

[18] Muzaffar U Fakiruddin KS Talebiashtiany Y Abdullah S . Delving deeper in the eye of the hurricane: Immunopathogenesis & molecular characterization of cytokine storm in COVID-19, association with disease severity & the therapeutic regimens. Cytokine. (2025) 195:157025. doi: 10.1016/j.cyto.2025.157025. PMID: 40945384

[19] Profir I Popescu CM Nechita A . Fatal influenza B-MRSA coinfection in a healthy adolescent: Necrotizing pneumonia, cytokine storm, and multi-organ failure. Children (Basel). (2025) 12:766. doi: 10.3390/children12060766. PMID: 40564724 PMC12191276

[20] Kang S Kishimoto T . Interplay between interleukin-6 signaling and the vascular endothelium in cytokine storms. Exp Mol Med. (2021) 53:1116–23. doi: 10.1038/s12276-021-00649-0. PMID: 34253862 PMC8273570

[21] Fajgenbaum DC June CH . Cytokine storm. N Engl J Med. (2020) 383:2255–73. doi: 10.1056/nejmra2026131. PMID: 33264547 PMC7727315

[22] Barrett D . IL-6 blockade in cytokine storm syndromes. Adv Exp Med Biol. (2024) 1448:565–72. doi: 10.1007/978-3-031-59815-9_37. PMID: 39117839

[23] Zegeye MM Lindkvist M Fälker K Kumawat AK Paramel G Grenegård M . Activation of the JAK/STAT3 and PI3K/AKT pathways are crucial for IL-6 trans-signaling-mediated pro-inflammatory response in human vascular endothelial cells. Cell Commun Signal. (2018) 16:55. doi: 10.1186/s12964-018-0268-4. PMID: 30185178 PMC6125866

[24] Kang S Tanaka T Inoue H Ono C Hashimoto S Kioi Y . IL-6 trans-signaling induces plasminogen activator inhibitor-1 from vascular endothelial cells in cytokine release syndrome. Proc Natl Acad Sci USA. (2020) 117:22351–6. doi: 10.1073/pnas.2010229117. PMID: 32826331 PMC7486751

[25] Kang SY Yoo JR Park Y Kim SH Heo ST Park SH . Fatal outcome of severe fever with thrombocytopenia syndrome (SFTS) and severe and critical COVID-19 is associated with the hyperproduction of IL-10 and IL-6 and the low production of TGF-β. J Med Virol. (2023) 95:e28894. doi: 10.22541/au.168112993.31971683/v1. PMID: 37386895

[26] Yoo JR Kim TJ Heo ST Hwang KA Oh H Ha T . IL-6 and IL-10 levels, rather than viral load and neutralizing antibody titers, determine the fate of patients with severe fever with thrombocytopenia syndrome virus infection in South Korea. Front Immunol. (2021) 12:711847. doi: 10.3389/fimmu.2021.711847. PMID: 34484214 PMC8416084

[27] Liu X Zhang F Qiao J He W . Serum IL-6 and IL-10 levels are associated with fatal outcomes in patients with SFTS in China. J Infect Dev Ctries. (2025) 19:273–9. doi: 10.3855/jidc.19939. PMID: 40063754

[28] Yoo JR Kim M Kang MJ Kim S Lee KH Heo ST . Tocilizumab for patients with severe fever with thrombocytopenia syndrome: Tocilizumab Observational SFTS Study-1. Yonsei Med J. (2025) 66:321–7. doi: 10.3349/ymj.2024.0209. PMID: 40288904 PMC12041401

[29] Guo Z Zhang Z Prajapati M Li Y . Lymphopenia caused by virus infections and the mechanisms beyond. Viruses. (2021) 13:1876. doi: 10.3390/v13091876. PMID: 34578457 PMC8473169

[30] Fathi N Rezaei N . Lymphopenia in COVID-19: therapeutic opportunities. Cell Biol Int. (2020) 44:1792–7. doi: 10.1002/cbin.11403. PMID: 32458561 PMC7283672

[31] Lalueza A Folgueira D Díaz-Pedroche C Hernández-Jiménez P Ayuso B Castillo C . Severe lymphopenia in hospitalized patients with influenza virus infection as a marker of a poor outcome. Infect Dis (Lond). (2019) 51:543–6. doi: 10.1080/23744235.2019.1598572. PMID: 31012776

[32] Qian F Zhou W Liu Y Ge Z Lai J Zhao Z . High C-reactive protein to lymphocyte ratio predicts mortality outcomes of patients with severe fever with thrombocytopenia syndrome: A multicenter study in China. J Med Virol. (2023) 95:e28546. doi: 10.1002/jmv.28546. PMID: 36734063

[33] Li MM Zhang WJ Weng XF Li MY Liu J Xiong Y . CD4 T cell loss and Th2 and Th17 bias are associated with the severity of severe fever with thrombocytopenia syndrome (SFTS). Clin Immunol. (2018) 195:8–17. doi: 10.1016/j.clim.2018.07.009. PMID: 30036637 PMC7185468

[34] Wang T Xu L Zhu B Wang J Zheng X . Immune escape mechanisms of severe fever with thrombocytopenia syndrome virus. Front Immunol. (2022) 13:937684. doi: 10.3389/fimmu.2022.937684. PMID: 35967309 PMC9366518

[35] Park A Park SJ Jung KL Kim SM Kim EH Kim YI . Molecular signatures of inflammatory profile and B-cell function in patients with severe fever with thrombocytopenia syndrome. mBio. (2021) 12:e02583-20. doi: 10.1128/mbio.02583-20. PMID: 33593977 PMC8545090

[36] Li MM Zhang WJ Liu J Li MY Zhang YF Xiong Y . Dynamic changes in the immunological characteristics of T lymphocytes in surviving patients with severe fever with thrombocytopenia syndrome (SFTS). Int J Infect Dis. (2018) 70:72–80. doi: 10.1016/j.ijid.2018.03.010. PMID: 29550447

[37] Li Z Hu J Bao C Gao C Zhang N Cardona CJ . Activation of the NLRP3 inflammasome and elevation of interleukin-1β secretion in infection by sever fever with thrombocytopenia syndrome virus. Sci Rep. (2022) 12:2573. doi: 10.21203/rs.3.rs-885550/v1. PMID: 35173184 PMC8850576

[38] Liu S Su Y Lu Z Zou X Xu L Teng Y . The SFTSV nonstructural proteins induce autophagy to promote viral replication via interaction with vimentin. J Virol. (2023) 97:e0030223. doi: 10.1128/jvi.00302-23. PMID: 37039677 PMC10134822

[39] Zhang L Fu Y Wang H Guan Y Zhu W Guo M . Severe fever with thrombocytopenia syndrome virus-induced macrophage differentiation is regulated by miR-146. Front Immunol. (2019) 10:1095. doi: 10.3389/fimmu.2019.01095. PMID: 31156641 PMC6529556

[40] Chen Y Miller H Benlagha K Byazrova MG Filatov AV Zhang Z . A focus on the mechanisms of alteration in host lymphocyte level following severe fever with thrombocytopenia syndrome virus (SFTSV) infection. J Inflammation Res. (2025) 18:13265–77. doi: 10.2147/jir.s531068. PMID: 41031180 PMC12477067

[41] Maeda K Malykhin A Teague-Weber BN Sun XH Farris AD Coggeshall KM . Interleukin-6 aborts lymphopoiesis and elevates production of myeloid cells in systemic lupus erythematosus-prone B6. Sle1. Yaa animals. Blood. (2009) 113:4534–40. doi: 10.1182/blood-2008-12-192559. PMID: 19224760 PMC2680362

[42] Jenkins BJ Roberts AW Greenhill CJ Najdovska M Lundgren-May T Robb L . Pathologic consequences of STAT3 hyperactivation by IL-6 and IL-11 during hematopoiesis and lymphopoiesis. Blood. (2007) 109:2380–8. doi: 10.1182/blood-2006-08-040352. PMID: 17082315

[43] Casel MA Park SJ Choi YK . Severe fever with thrombocytopenia syndrome virus: Emerging novel phlebovirus and their control strategy. Exp Mol Med. (2021) 53:713–22. doi: 10.1038/s12276-021-00610-1. PMID: 33953322 PMC8178303

[44] Sun Y Jin C Zhan F Wang X Liang M Zhang Q . Host cytokine storm is associated with disease severity of severe fever with thrombocytopenia syndrome. J Infect Dis. (2012) 206:1085–94. doi: 10.1093/infdis/jis452. PMID: 22904342

