# Supplementary material for: Interleukin-6-to-lymphocyte ratio as a novel prognostic factor in patients with severe fever with thrombocytopenia syndrome: a discovery and validation study
Source: Front Immunol. 2026 Mar 25;17:1780148. doi: 10.3389/fimmu.2026.1780148 (PMC13057444; doi:10.3389/fimmu.2026.1780148)
Supplement: Supplementary file 1 [file Table1.docx]

Supplementary Material

# Supplementary Tables

**Supplementary Table 1. Baseline characteristics of the training and validation cohorts of SFTS patients**

| **Characteristics** | **Total**  **(n = 495)** | **Training cohort**  **(n = 323)** | **Validation cohort**  **(n = 172)** | ***P* value** |
| --- | --- | --- | --- | --- |
| Age, year | 67.00  (60.00, 74.00) | 67.00  (59.00, 74.00) | 68.00  (60.00, 75.00) | 0.173 |
| Gender, (male), n (%) | 218 (44.04) | 149 (46.13) | 69 (40.12) | 0.199 |
| Days from symptom onset | 5.00 (4.00, 7.00) | 5.00 (4.00, 7.00) | 6.00 (4.00, 7.00) | 0.170 |
| **Comorbidities, n (%)** | | | | |
| Diabetes | 76 (15.35) | 52 (16.10) | 24 (13.95) | 0.528 |
| Hypertension | 129 (26.06) | 88 (27.24) | 41 (23.84) | 0.411 |
| Cardiovascular disease | 27 (5.45) | 11 (3.41) | 16 (9.30) | **0.006** |
| Cerebrovascular disease | 23 (4.65) | 16 (4.95) | 7 (4.07) | 0.656 |
| **Symptoms, n (%)** | | | | |
| Fever | 472 (95.35) | 306 (94.74) | 166 (96.51) | 0.371 |
| Shiver | 72 (14.55) | 53 (16.41) | 19 (11.05) | 0.107 |
| Fatigue | 429 (86.67) | 274 (84.83) | 155 (90.12) | 0.099 |
| Inappetence | 379 (76.57) | 267 (82.66) | 112 (65.17) | **< 0.001** |
| Nausea | 250 (50.51) | 155 (47.99) | 95 (55.23) | 0.125 |
| Vomit | 144 (29.09) | 91 (28.17) | 53 (30.81) | 0.538 |
| Abdominal pain | 50 (10.10) | 31 (9.60) | 19 (11.05) | 0.610 |
| Diarrhea | 168 (33.94) | 123 (38.08) | 45 (26.16) | **0.008** |
| Chest distress | 32 (7.07) | 22 (6.81) | 10 (5.81) | 0.667 |
| Palpitation | 23 (4.65) | 15 (4.64) | 8 (4.65) | 0.997 |
| Myalgia | 182 (36.77) | 117 (36.22) | 65 (37.79) | 0.730 |
| **Signs, n (%)** | | | | |
| Hemorrhage | 64 (12.93) | 45 (13.93) | 19 (11.05) | 0.362 |
| Lymphadenectasis | 129 (26.06) | 103 (31.89) | 26 (15.12) | **< 0.001** |
| Neurological signs | 167 (33.74) | 86 (26.63) | 81 (47.09) | **< 0.001** |
| **Laboratory parameters** | | | | |
| DBV RNA (log_10_ TCID50/mL) | 3.23 ± 1.58 | 3.29 ± 1.53 | 3.10 ± 1.65 | 0.187 |
| WBC (10^9^/L) | 2.34 (1.62, 3.71) | 2.16 (1.56, 3.49) | 2.79 (1.82, 4.95) | **< 0.001** |
| Neutrophils (10^9^/L) | 1.41 (0.90, 2.45) | 1.30 (0.88, 2.10) | 1.57 (0.97, 3.19) | **0.007** |
| Lymphocytes (10^9^/L) | 0.55 (0.36, 0.93) | 0.46 (0.32, 0.77) | 0.75 (0.45, 1.31) | **< 0.001** |
| Monocytes (10^9^/L) | 0.16 (0.08, 0.36) | 0.15 (0.08, 0.35) | 0.19 (0.07, 0.39) | 0.843 |
| HGB (g/L) | 141.00 (128.00, 151.00) | 144.00 (132.00, 154.00) | 137.50 (123.00, 147.00) | **< 0.001** |
| PLT (10^9^/L) | 62.00 (45.00, 86.00) | 67.00 (49.00, 88.00) | 55.50 (40.00, 80.75) | **0.001** |
| ALT (U/L) | 64.00 (39.80, 114.50) | 62.40 (39.10, 114.50) | 64.00 (40.25, 118.25) | 0.733 |
| AST (U/L) | 128.00 (68.20, 268.30) | 128.00 (68.60, 268.10) | 128.50 (66.25, 279.25) | 0.758 |
| ALB (g/L) | 32.53 ± 4.75 | 32.59 ± 4.89 | 32.43 ± 4.49 | 0.730 |
| CK (U/L) | 391.00 (171.00, 925.00) | 449.00 (191.00, 1133.00) | 317.50 (137.25, 773.00) | **0.006** |
| LDH (U/L) | 545.00 (354.00, 918.00) | 549.00 (374.00, 932.00) | 541.00 (307.50, 885.25) | 0.321 |
| UREA (mmol/L) | 5.84 (4.28, 7.97) | 5.95 (4.51, 8.37) | 5.53 (4.02, 7.29) | **0.014** |
| CREA (umol/L) | 65.80 (52.70, 83.20) | 67.00 (53.30, 84.20) | 63.44 (50.83, 80.23) | 0.070 |
| PT (sec) | 12.70 (12.10, 13.40) | 13.00 (12.50, 13.70) | 11.85 (11.20, 12.60) | **< 0.001** |
| APTT (sec) | 46.30 (38.50, 53.10) | 48.80 (42.60, 56.20) | 38.85 (33.78, 46.23) | **< 0.001** |
| FIB (g/L) | 2.45 (2.08, 2.85) | 2.56 (2.15, 2.93) | 2.26 (1.93, 2.65) | **< 0.001** |
| Hs-CRP (mg/L) | 3.20 (0.93, 9.86) | 3.20 (1.09, 8.94) | 3.17 (0.76, 12.20) | 0.748 |
| PCT (ng/ml) | 0.17 (0.09, 0.42) | 0.16 (0.09, 0.44) | 0.18 (0.09, 0.36) | 0.868 |
| IL-6 (log_10_ pg/ml) | 1.45 ± 0.73 | 1.37 ± 0.73 | 1.60 ± 0.69 | **0.001** |
| IL-10 (log_10_ pg/ml) | 1.18 ± 0.68 | 1.12 ± 0.67 | 1.28 ± 0.69 | **0.017** |
| IFN-γ (log_10_ pg/ml) | 2.03 (1.37, 2.44) | 2.05 (1.27, 2.52) | 2.00 (1.43, 2.33) | 0.060 |
| TNF-α (log_10_ pg/ml) | 0.28 (0.00, 0.52) | 0.23 (0.00, 0.34) | 0.59 (-0.11, 1.07) | **< 0.001** |
| IL-6/LY (log_10_) | 1.68 ± 0.84 | 1.67 ± 0.84 | 1.72 ± 0.85 | 0.535 |

Abbreviations: SFTS: severe fever with thrombocytopenia syndrome, DBV: Bandavirus dabieense, WBC: white blood cell, HGB: hemoglobin, PLT: platelet, ALT: alanine aminotransaminase, AST: aspartate aminotransferase, ALB: albumin, CK: creatine phosphokinase, LDH: lactate dehydrogenase, CREA: creatinine, PT: prothrombin time, APTT: activated partial thromboplastin time, FIB: fibrinogen, Hs-CRP: high-sensitivity C-reactive protein, PCT: procalcitonin, IL-6: interleukin-6, IL-10: interleukin-10, IFN-γ: interferon-gamma, TNF-α: tumor necrosis factor-alpha, IL-6/LY: interleukin-6-to-lymphocyte ratio.

Continuous variable data are presented as mean (SD), median (interquartile ranges, IQR). Classified variable data are presented as n (%). P values indicate differences between the training and validation cohorts.

*P* < 0.05 was considered statistically significant. Statistically significant values are shown in bold.

**Supplementary Table 2. Clinical and laboratory characteristics of survivors and non-survivors in the validation cohort of SFTS patients**

| **Characteristics** | **Total**  **(n = 172)** | **Survivors**  **(n = 134)** | **Non-Survivors**  **(n = 38)** | ***P* value** |
| --- | --- | --- | --- | --- |
| Age, year | 68.00  (60.00, 75.00) | 67.00  (60.00, 73.00) | 72.50  (65.75, 77.00) | **0.006** |
| Gender, (male), n (%) | 69 (40.12) | 58 (43.28) | 11 (28.95) | 0.112 |
| Days from symptom onset | 6.00 (4.00, 7.00) | 6.00 (4.00, 7.00) | 5.00 (4.00, 7.00) | 0.217 |
| **Comorbidities, n (%)** | | | | |
| Diabetes | 24 (13.95) | 13 (9.70) | 11 (28.95) | **0.003** |
| Hypertension | 41 (23.84) | 35 (26.12) | 6 (15.79) | 0.187 |
| Cardiovascular disease | 16 (9.30) | 11 (8.21) | 5 (13.16) | 0.354 |
| Cerebrovascular disease | 7 (4.07) | 6 (4.48) | 1 (2.63) | 0.611 |
| **Symptoms, n (%)** | | | | |
| Fever | 166 (96.51) | 128 (95.52) | 38 (100.00) | 0.184 |
| Shiver | 19 (11.05) | 13 (9.70) | 6 (15.79) | 0.291 |
| Fatigue | 155 (90.12) | 123 (91.79) | 32 (84.21) | 0.167 |
| Inappetence | 112 (65.12) | 91 (67.91) | 21 (55.26) | 0.149 |
| Nausea | 95 (55.23) | 79 (58.96) | 16 (42.11) | 0.065 |
| Vomit | 53 (30.81) | 42 (31.34) | 11 (28.95) | 0.778 |
| Abdominal pain | 19 (11.05) | 15 (11.19) | 4 (10.53) | 0.908 |
| Diarrhea | 45 (26.16) | 36 (26.87) | 9 (23.68) | 0.694 |
| Chest distress | 10 (5.81) | 5 (3.73) | 5 (13.16) | **0.028** |
| Palpitation | 8 (4.65) | 5 (3.73) | 3 (7.89) | 0.282 |
| Myalgia | 65 (37.79) | 54 (40.30) | 11 (28.95) | 0.203 |
| **Signs, n (%)** | | | | |
| Hemorrhage | 19 (11.05) | 10 (7.46) | 9 (23.68) | **0.005** |
| Lymphadenectasis | 26 (15.12) | 21 (15.67) | 5 (13.16) | 0.703 |
| Neurological signs | 81 (47.09) | 46 (34.33) | 35 (92.11) | **< 0.001** |
| **Laboratory parameters** | | | | |
| DBV RNA (log10 TCID50/mL) | 3.10 ± 1.65 | 2.61 ± 1.46 | 4.83 ± 0.98 | **< 0.001** |
| WBC (10^9^/L) | 2.79 (1.82, 4.95) | 2.79 (1.72, 5.06) | 2.82 (2.07, 4.69) | 0.856 |
| Neutrophils (10^9^/L) | 1.57 (0.97, 3.19) | 1.45 (0.92, 2.97) | 2.09 (1.42, 3.63) | **0.039** |
| Lymphocytes (10^9^/L) | 0.75 (0.45, 1.31) | 0.85 (0.49, 1.39) | 0.59 (0.38, 0.76) | **0.003** |
| Monocytes (10^9^/L) | 0.19 (0.07, 0.39) | 0.21 (0.09, 0.40) | 0.08 (0.04, 0.20) | **< 0.001** |
| HGB (g/L) | 137.50 (123.00, 147.00) | 137.00 (122.75, 147.00) | 138.50 (122.25, 149.50) | 0.666 |
| PLT (10^9^/L) | 55.50 (40.00, 80.75) | 59.00 (42.75, 86.25) | 46.50 (30.50, 56.75) | **0.002** |
| ALT (U/L) | 64.00 (40.25, 118.25) | 59.00 (35.75, 105.50) | 77.50 (55.25, 208.00) | **0.010** |
| AST (U/L) | 128.50 (66.25, 279.25) | 95.00 (62.75, 233.25) | 290.00 (150.75, 704.00) | **< 0.001** |
| ALB (g/L) | 32.43 ± 4.49 | 33.10 ± 4.38 | 30.07 ± 4.14 | **< 0.001** |
| CK (U/L) | 317.50 (137.25, 773.00) | 267.00 (122.75, 575.00) | 852.00 (344.00, 1842.25) | **< 0.001** |
| LDH (U/L) | 541.00 (307.50, 885.25) | 471.00 (289.00, 781.50) | 1130.00 (615.25, 1599.75) | **< 0.001** |
| UREA (mmol/L) | 5.53 (4.02, 7.29) | 5.17 (3.87, 6.55) | 7.52 (5.07, 12.79) | **< 0.001** |
| CREA (umol/L) | 63.44 (50.83, 80.23) | 61.20 (49.85, 74.63) | 73.50 (52.45, 115.85) | **0.010** |
| PT (sec) | 11.85 (11.20, 12.60) | 11.75 (11.20, 12.40) | 12.60 (11.55, 14.10) | **0.001** |
| APTT (sec) | 38.85 (33.78, 46.23) | 37.15 (32.18, 42.75) | 49.20 (42.35, 59.08) | **< 0.001** |
| FIB (g/L) | 2.26 (1.93, 2.65) | 2.26 (1.98, 2.70) | 2.11 (1.81, 2.54) | 0.127 |
| Hs-CRP (mg/L) | 3.17 (0.76, 12.20) | 2.30 (0.47, 7.29) | 13.52 (7.51, 31.42) | **< 0.001** |
| PCT (ng/ml) | 0.18 (0.09, 0.36) | 0.13 (0.08, 0.26) | 0.47 (0.20, 1.49) | **< 0.001** |
| IL-6 (log_10_ pg/ml) | 1.60 ± 0.69 | 1.45 ± 0.67 | 2.12 ± 0.48 | **< 0.001** |
| IL-10 (log_10_ pg/ml) | 1.28 ± 0.69 | 1.17 ± 0.69 | 1.67 ± 0.53 | **< 0.001** |
| IFN-γ (log_10_ pg/ml) | 2.00 (1.43, 2.33) | 1.90 (1.31, 2.28) | 2.30 (1.68, 2.55) | **0.002** |
| TNF-α (log_10_ pg/ml) | 0.59 (-0.11, 1.07) | 0.47 (-0.12, 1.04) | 0.86 (0.25, 1.12) | **0.026** |
| IL-6/LY (log_10_) | 1.72 ± 0.85 | 1.53 ± 0.81 | 2.37 ± 0.62 | **< 0.001** |

Abbreviations: SFTS: severe fever with thrombocytopenia syndrome, DBV: Bandavirus dabieense, WBC: white blood cell, HGB: hemoglobin, PLT: platelet, ALT: alanine aminotransaminase, AST: aspartate aminotransferase, ALB: albumin, CK: creatine phosphokinase, LDH: lactate dehydrogenase, CREA: creatinine, PT: prothrombin time, APTT: activated partial thromboplastin time, FIB: fibrinogen, Hs-CRP: high-sensitivity C-reactive protein, PCT: procalcitonin, IL-6: interleukin-6, IL-10: interleukin-10, IFN-γ: interferon-gamma, TNF-α: tumor necrosis factor-alpha, IL-6/LY: interleukin-6-to-lymphocyte ratio.

Continuous variable data are presented as mean (SD), median (interquartile ranges, IQR). Classified variable data are presented as n (%). P values indicate differences between survivors and non-survivors in the validation cohort.

*P* < 0.05 was considered statistically significant. Statistically significant values are shown in bold.

**Supplementary Table 3. Collinearity diagnostics of variables before multivariate logistic regression analysis in patients with SFTS in the training cohort**

| **Parameters** | **Tolerance** | **VIF** |
| --- | --- | --- |
| Age | 0.908 | 1.102 |
| DBV RNA | 0.500 | 1.999 |
| PLT | 0.782 | 1.279 |
| UREA | 0.764 | 1.310 |
| AST | 0.729 | 1.371 |
| PT | 0.855 | 1.170 |
| IL-6/LY | 0.558 | 1.792 |

Abbreviations: SFTS: severe fever with thrombocytopenia syndrome, DBV: Bandavirus dabieense, PLT: platelet, AST: aspartate aminotransferase, PT: prothrombin time, IL-6/LY: interleukin-6-to-lymphocyte ratio, VIF: variance inflation factor.

**Supplementary Table 4. Comparison of AUCs of IL-6/LY with IL-6 and lymphocytes in the training cohort of SFTS patients based on DeLong’s test**

| **Comparison** | **AUC (95% CI)** | ***P*** |
| --- | --- | --- |
| IL-6/LY vs. IL-6 | 0.839 (0.792–0.886) vs. 0.828 (0.778–0.878) | 0.282 |
| IL-6/LY vs. Lymphocytes | 0.839 (0.792–0.886) vs. 0.628 (0.555–0.700) | **< 0.001** |

Abbreviations: SFTS: severe fever with thrombocytopenia syndrome, AUC: area under the curve, IL-6: interleukin-6, IL-6/LY: interleukin-6-to-lymphocyte ratio, 95% CI: 95% confidence interval.

*P* < 0.05 was considered statistically significant. Statistically significant values are shown in bold.

**Supplementary Table 5. Comparison of AUCs of IL-6/LY with IL-6 and lymphocytes in the validation cohort of SFTS patients based on DeLong’s test**

| **Comparison** | **AUC (95% CI)** | ***P*** |
| --- | --- | --- |
| IL-6/LY vs IL-6 | 0.812 (0.735–0.890) vs. 0.805 (0.729–0.881) | 0.701 |
| IL-6/LY vs Lymphocytes | 0.812 (0.735–0.890) vs. 0.660 (0.566–0.753) | **< 0.001** |

Abbreviations: SFTS: severe fever with thrombocytopenia syndrome, AUC: area under the curve, IL-6: interleukin-6, IL-6/LY: interleukin-6-to-lymphocyte ratio, 95% CI: 95% confidence interval.

*P* < 0.05 was considered statistically significant. Statistically significant values are shown in bold.

**Supplementary Table 6. Incremental predictive value of IL-6/LY for mortality in SFTS patients**

| **Cohort** | **Model** | **NRI (95% CI)** | ***P* value** | **IDI (95% CI)** | ***P* value** |
| --- | --- | --- | --- | --- | --- |
| **Training Cohort** | Model 1 | Reference | NA | Reference | NA |
|  | Model 2 | 0.676 (0.419–0.933) | **< 0.001** | 0.063 (0.029–0.097) | **< 0.001** |
| **Validation Cohort** | Model 1 | Reference | NA | Reference | NA |
|  | Model 2 | 0.795 (0.479–1.111) | **< 0.001** | 0.075 (0.022–0.129) | **0.006** |
| **Combined Cohort** | Model 1 | Reference | NA | Reference | NA |
|  | Model 2 | 0.732 (0.531–0.933) | **< 0.001** | 0.066 (0.038–0.094) | **< 0.001** |

Abbreviations: SFTS: severe fever with thrombocytopenia syndrome, NRI: net reclassification improvement, IDI: integrated discrimination improvement, 95% CI: 95% confidence interval, IL-6/LY: interleukin-6-to-lymphocyte ratio, DBV: Bandavirus dabieense.

Model 1: age + DBV RNA. Model 2: Model 1 + IL-6/LY. NRI refers to continuous NRI.

NA, not applicable (Model 1 served as the reference model, and no P value was calculated).

*P* < 0.05 was considered statistically significant. Statistically significant values are shown in bold.
